# Peritonitis from Perforation of the Appendix Vermiformis

**Published:** 1842-10-08

**Authors:** 


*Peritonitis from Perforation of the Appendix Vermiformis.*—T. Kennell, æt. 18, entered the University College Hospital, June 21, 1842, under the care of Dr. Taylor. He is of the ordinary conformation and florid complexion; he is a shoemaker, and single: always had enough to eat, and always lived regularly and temperately; never been intoxicated; resides in a dry but confined situation; parents dead, does not know of what they died. He generally enjoys good health, and thinks he has an excellent constitution. About eight years ago, he states that he had an inflammation of the left chest accompanied with pleurisy. He does not recollect coughing or spitting at the time; he had shortness of breath, severe short pain below the ribs, and not above the margin of the lower ribs. He was bled, leeched, and blistered; has had no complaint since. The present attack commenced on Sunday, June 19, at 3 A. M., suddenly, with severe sharp pain all the over the abdomen, with sickness, vomiting, and some diarrhœa; no rigor or feverishness preceded the pain, which has continued to increase until the present time; it is constant and unremitting, but increased at times, and upon motion, taking a deep breath, and upon pressure, but not by pinching; it extends all over the abdomen, but is worse on the right side of it and in the hypogastrium; the sickness and vomiting are much better than they were at first; the matter vomited is of a deep green colour. He has shortness of breath on account of the pain in the abdomen; his breathing is entirely costal. His bowels have not been open since Friday until this morning, when they were opened freely; the motion was of a white colour, and very watery. Yesterday he took ninepenny-worth of castor oil, four doses of a mixture, a powder and two pills, and applied sixteen leeches to the abdomen last night. The leeches did not afford him any relief. Tongue moist, covered with a light, brown fur; papillæ at the apex more distinct than usual; great thirst; skin hot; countenance flushed, but very little anxiety ; pulse 116, full, moderately firm, and rather jerking; urine scanty, high-coloured, when passing it causes pain in the abdomen. He lies on his back with his knees drawn up. The abdomen seems moderately distended, and yields a tympanitic sound on slight percussion; heart's impulse a little strong, no murmurs. He has not eaten or drunk anything but his usual diet, and has followed his employment as usual. He was ordered to be bled to eighteen ounces, to have three grains of calomel every three hours, and to have the abdomen fomented with turpentine.

June 22. Dr. Quain saw him at eight yesterday evening, when he felt somewhat better. He was ordered an injection, consisting of two ounces of starch with forty drops of tincture of opium ; this gave him great pain. His bowels were opened several times before the injection, but have not been since. Dr. Quain saw him again at six this morning : his pulse was 160; abdomen more full, and felt doughy and firm. Ordered beef-tea. He did not sleep above ten minutes in the night; felt more pain in the night than previously, and was very feverish. This morning, at ten, he has not much pain: still lies on his back, with his legs drawn up ; somewhat restless; countenance anxious ; great thirst ; vomits frequently a matter of brown color, having no odour; extremities cold ; general surface of body cadaverous. Died at twenty minutes to twelve to-day.

*After-death Appearances.*

The body was examined twenty-four hours after death. Abdomen much distended, and tympanitic. On cutting into the peritoneum some turbid, fœtid serum escaped ; there was pus mixed with the serum. The peritoneum redder on the right side than the left. The scum in the neighbourhood of the gall-bladder yellow. Intestines matted together by recent adhesion, and much distended. The peritoneum adherent to the gall-bladder. The redness ceases where the intestines are agglutinated, but passes over; mesentery much thickened and softened; stomach marbled at the pyloric orifice with black colouring matter, much reddened at the cul de sac; mucous membrane not softened. In the large intestines the solitary glands are very distinct and elevated; but in the small intestines, they and Peyer's glands were scarcely visible. The vermiform appendix dark coloured, with thickened coats. Two perforations with rounded edges were seen. Mucous membrane throughout inflamed, and riddled with ulcerations; the exterior was also much inflamed. A gritty brown substance, about the size of a bean, was found in it. On a section a nucleus was found, the matter surrounding the nucleus appeared to be fæcal.

*Chest.*—The whole of the left lung was found adherent to the walls of the chest by firm old adhesions ; about two ounces of bloody serum in the right chest; right lung, of a deep red colour, crepitates freely, very lacerable at the back part; pleura of the left lung adherent to the pericardium. The whole of the left lung smaller and firmer than usual, crepitates less than the right; lower lobe lacerable; pericardium loosely adherent to the heart by old adhesions. Heart covered with much fat; surface redder than usual; firm coagulum, partly fibrinous, in the right auricle and ventricle. Cavity of the left ventricle natural in size; endocardium thickened and opake; walls darker coloured than usual, and fully as thick; mitral valve admits two fingers. Walls of the right ventricle thicker than usual; endocardium thick and opake. Nothing unusual was observed in other organs.—*Lond. Lancet*, August 20, 1842.

